# Versatility of Pedicled Tensor Fascia Lata Flap: A Useful and Reliable Technique for Reconstruction of Different Anatomical Districts

**DOI:** 10.1155/2014/846082

**Published:** 2014-11-18

**Authors:** Md. Sohaib Akhtar, Mohd Fahud Khurram, Arshad Hafeez Khan

**Affiliations:** Post Graduate Department of Burns, Plastic and Reconstructive Surgery, JNMC, AMU, Aligarh, India

## Abstract

*Aims and Objectives*. The aim of this study was to evaluate the versatility of pedicled tensor fascia lata flap for reconstruction of various anatomical regions. *Materials and Methods*. In this retrospective study a total of 34 patients with defects over various anatomical regions were included. The defects were located over the trochanter (*n* = 12), groin (*n* = 8), perineum (*n* = 6), lower anterior abdomen (*n* = 6), gluteal region (*n* = 1), and ischial region (*n* = 1). The etiology of defects included trauma (*n* = 12), infection (*n* = 8), pressure sores (*n* = 8), and malignancy (*n* = 6). Reconstruction was performed using pedicled tensor fascia lata flaps. Patients were evaluated in terms of viability of the flap and donor site morbidity. The technical details of the operative procedure have also been outlined. *Results*. All the flaps survived well except 5 patients in which minor complications were noted and 1 who experienced complete flap loss. Of those with minor complications, 1 patient developed distal marginal necrosis and 1 developed infection which subsided within three days by dressings and antibiotics and in 2 patients partial loss of the skin graft occurred at the donor site out of which 1 required regrafting and another one healed completely with dressing and antibiotics. All the patients were followed up for an average period of 6 months, ranging from 1 to 12 months. Donor site morbidity was minimal. *Conclusion*. It was concluded that the pedicled tensor fascia lata flap is a versatile, reliable, easy, and less time consuming procedure for the coverage of defects around trochanter, groin, lower anterior abdomen, perineum, and ischial region.

## 1. Introduction

Wangensteen first reported the tensor fascia lata flap for abdominal wall reconstruction [1]. Later, in 1978, Hill et al. and Nahai et al. described it as free musculocutaneous flap. Since then it has widely been used for reconstruction of various anatomical regions as pedicled or free flaps [2, 3].

This is a myofasciocutaneous flap that can be used as a pedicled flap for a wide variety of regions including trochanter, groin, perineum, ischium, and lower abdomen that can occur following trauma, infection, orthopaedic intervention, and pressure sores and after resection of malignant lesions/lymph node dissection.

These regions are usually associated with exposure of vital structures including bone or vessels. Therefore these anatomical districts almost always require flap cover.

Currently there are many options available for the reconstruction of these regions. Tensor fascia lata flap is one of the good alternatives.

Due to various features of tensor fascia lata flap, it is considered as a reliable flap for the reconstruction of many challenging defects [4, 5]. This includes the fact that this flap can be used as motor and sensory innervated; it has adequate and different types of soft tissues [4–7].

In this series we present our experience with the use of pedicled tensor fascia lata flap for the reconstruction of various anatomical regions.

## 2. Materials and Methods

In this retrospective study a total of 34 patients with defects over various anatomical regions were included. The defects were located over the trochanter (*n* = 12), groin (*n* = 8), perineum (*n* = 6), lower anterior abdomen (*n* = 6), gluteal region (*n* = 1), and ischial region (*n* = 1). The etiology of defects included trauma (*n* = 12), infection (*n* = 8), pressure sores (*n* = 8), and malignancy (*n* = 6). Reconstruction was performed using pedicled tensor fascia lata flaps. The technical details and operative time of the procedure have also been outlined.

All of the flaps were harvested from the lateral thigh regions and the raw area created was covered with a split thickness skin graft or closed primarily. The size of the flaps was slightly bigger to those of the defects. All flaps were based on a single dominant pedicle of ascending branch of lateral circumflex artery. The following operative steps were performed.

1. First of all, anterior border of the flap was delineated by drawing a line from anterior superior iliac spine and lateral condyle of tibia.
2. The posterior border of the flap was represented by greater trochanter, superior border by iliac crest, and inferior border within 8 cm from joint line.
3. Location of perforators was marked with hand held doppler at the junction of proximal and middle third.
4. Recipient site was prepared and actual size of the defect was measured.
5. Size and location of the flap was designed by following principle of planning in reverse.
6. Lower border of the flap was incised first followed by anterior and posterior border.
7. Dissection was performed in distal to proximal direction in the subfascial plane.
8. The vascular pedicle was identified at the preoperatively marked site at anterior aspect (8–10 cm distal to anterior superior iliac spine).
9. Flap was then transferred to the recipient site and finally in setting and suturing of the flap was done into the defect.
10. Donor site was skin grafted or primarily closed.

## 3. Results

Patients were evaluated in terms of viability of the flap and donor site morbidity. All the flaps survived well except 5 patients in which minor complications were noted and 1 who experienced complete flap loss.

Of those with minor complications, 1 patient developed distal marginal necrosis and 1 developed infection which subsided within three days by dressings and antibiotics and in 2 patients partial loss of the skin graft occurred at the donor site out of which 1 required regrafting and another one healed completely with dressing and antibiotics.

The length of the flap ranged from 14 to 22 cm and the width from 6 to 12 cm with mean operating time 1 hr.

Out of thirty-four patients, 28 were males and 6 were females. The average age of the patients was 30.4 years (range, 14 to 58 years).

All the patients were followed up for an average period of 6 months, ranging from 1 to 12 months. Donor site morbidity was minimal. No newly developed functional deficit of the lower limb was noted in any patient.

## 4. Discussion

Reconstruction of soft tissue defects around trochanter, groin, perineum, ischium, and lower abdomen remains a challenging task for the plastic surgeon. These regions often require flap cover due to associated exposure of bones or vessels or other vital structures. The flap can be harvested from local or remote areas. The local flaps can be taken from abdomen or thigh. Many local flaps have been described in the literature.

The various muscle flaps used for groin regions are rectus abdominis flap [8], rectus femoris flap, sartorius flap, internal oblique flap, and vastus lateralis flap [9–11].

For lower abdominal wall reconstruction, the available options are rectus abdominis muscle flap [12], tissue expansion [13], and medial mobilization of abdominal wall muscle [14].

Similarly, for perineal defects, the various reconstructive options are gracilis myocutaneous flap [15], rectus abdominis flaps [16], deep inferior epigastric perforator (DIEP) flap [17], superior gluteal artery perforator (SGAP) flap [18], and gluteal (split) flaps [19].

For ischial regions, gluteus maximus muscle flap [20], myocutaneous or fasciocutaneous posterior thigh flap [21], and gracilis muscle flap [22] have been well described in the literature.

Out of these numerous surgical techniques used for reconstruction of above-mentioned anatomical districts, each has its own advantages and disadvantages.

Use of rectus abdominis muscle flap may lead to abdominal weakness [11] or hernia, rectus femoris may cause knee weakness [23], and gluteus maximus may lead to gait disturbance [24].

Size of the internal oblique muscle is small and its dissection can be difficult and bloody [9].

Medial mobilization of abdominal muscle is difficult to use in the field of surgical oncology and tissue expansion takes a longer time [25]. Functional deficit may follow with the use of gracilis muscle flap.

The tensor fascia lata flap is a reliable flap having good vascularity and composed of skin, subcutaneous tissue, fascia, and muscle. Besides the fact that that this muscle is expendable, it causes minimal donor site morbidity without any knee weakness [26].

In this study we have used tensor fascia lata flap in different anatomical soft tissue defects. We observed a high success rate of this flap. All the flaps survived well except 5 patients in which minor complications were noted and 1 who experienced complete flap loss.

**Figure 1:**
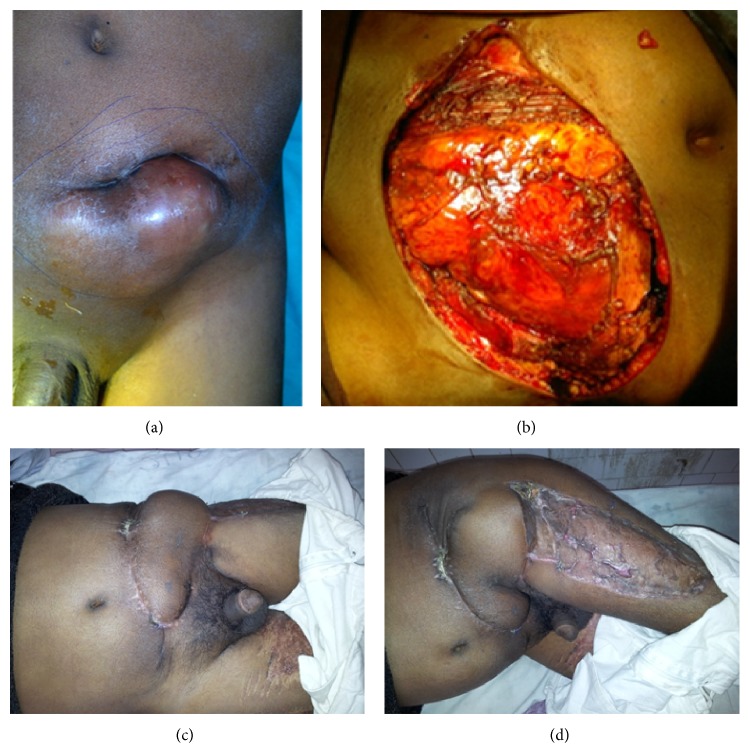
(a) Left lower abdominal wall malignancy (dermatofibrosarcoma). (b) Defect after local wide excision of tumour. (c) Follow-up photograph showing well set flap. (d) Follow-up photograph showing well set flap and well taken-up skin graft.

Six flaps in this series were used for abdominal wall reconstruction (Figure 1). We found no complication in terms of herniation or abdominal wall weakness. This corresponds to literature as described by some authors who reported that fascia lata is an effective flap that avoids the use of mesh and having a low incidence of recurrent herniation [27, 28].

We performed groin reconstruction in 8 patients, out of which 4 underwent lymph node dissection due to malignancy in different regions, 2 had traumatic soft tissue loss, and 2 had postinfectious soft tissue loss.

Agarwal et al. [29] performed pedicled tensor fascia lata thigh flap after block dissection of inguinal lymph nodes for malignant deposits in 15 patients. They observed that there were two cases of marginal flap necrosis, three cases developed lymphoedema which was managed by stockings, there were two cases of infection which were settled by antibiotics, and there were three cases of loss of a small area of skin graft at the donor site. They concluded that pedicled tensor fascia lata flap is a good and reliable option for groin reconstruction.

**Figure 2:**
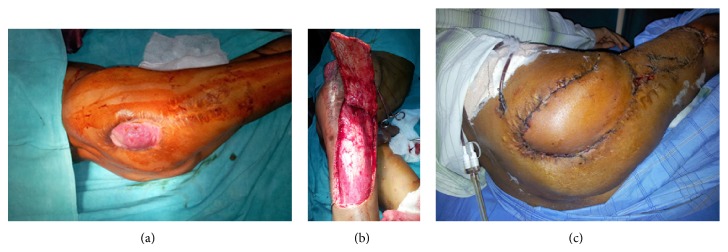
(a) Postarthroplasty soft tissue defect over right trochanteric region. (b) Photograph showing TFL flap elevation and arc of rotation of the flap. (c) Postoperative photograph after 2 weeks showing well set flap and well taken-up graft at donor site.

**Figure 3:**
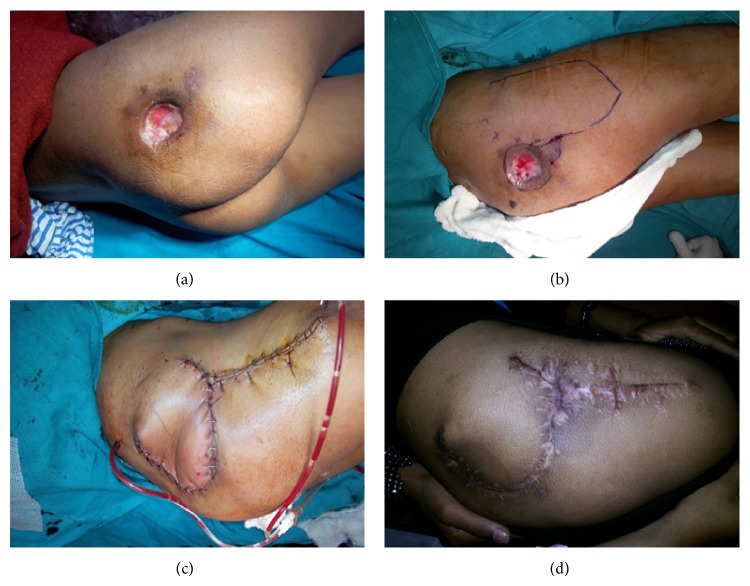
(a) Postinfection soft tissue defect over right gluteal region. (b) Photograph showing markings and TFL flap design. (c) Immediate postoperative photograph showing flap inset into the defect and donor site primarily closed. (d) Long term follow-up photograph.

Pedicled tensor fascia lata flap was performed in majority of the patients with defects over trochanter totalling around 30% (12 patients) (Figures 2 and 3).

Karabeg et al. [30] used 39 pedicled TFL flaps for reconstruction of trochanteric pressure sore defects in 34 patients. The size of the flaps used ranged from 15 × 6 cm to 30 × 15 cm. All flaps survived with distal tip necrosis occurred in 4 cases where very large flap was used beyond the safe limit. They found that tensor fascia lata flap is reliable flap but problem with the flap can be encountered if the flap is harvested beyond the safe limits and improperly designed. Only 1 patient in our study underwent distal tip necrosis. The reason of low complication is that we kept the size of the flap within the safe limit.

One of the good alternatives for ischial sore reconstruction is tensor fascia lata flap [3]. We used TFL flap in 1 patient in ischial sore reconstruction. All flaps survived well.

For perineal reconstruction, TFL can be safely used for lateral defects including inguinal regions [2, 31]. The size and consequently reach of the flap can be increased when used in combination with anterolateral thigh flap [32].

A total of 6 patients with perineal defects were included in this study, 2 with defects in the medial side and 4 having lateral defects. In those with medial defects TFL was used in combination with ALT flap. One flap in this group sustained complete necrosis. Compression or twisting of the pedicle could be the possible reason of total loss of the flap.

Contedini et al. [33] reported 11 cases of soft tissue reconstruction firstly planned with the ALT flap and then converted into TFL perforator flap. They concluded that result was always satisfactory in terms of the donor site morbidity and reconstructive outcome. The main disadvantage of anterolateral thigh flap is its anatomical variability in number and location of perforators. They found that TFL perforator flap can be a good alternative. Some other researchers reported the similar possibility [34, 35]. The anatomy of TFL is more constant than ALT flap [36].

Aesthetic problem at the donor site could be avoided by using free flaps. However free flaps have their own limitations including high anaesthesia risk, long operating time, demands of microsurgical expertise, and a costly well equipped microsurgical set-up. Besides that, we have limited facility of microsurgery at our centre.

Main advantage of this flap is that it is very fast and reliable, so it is a good technique for critical patients who require a fast operation and cannot tolerate a lengthy operation that may lead to higher rate of complication. Other advantage of TFL flap is that it consist of highly vascular skin, subcutaneous tissue, fascia, and muscle and can be used to cover even irradiated tissues. Other advantage of this flap is that the donor site grafted site is hidden and less visible as compared to other donor sites from abdomen and groin thus minimising aesthetic concerns.

We acknowledge the limitation of our study. In some instances, TFL was used despite the availability of other options.

## 5. Conclusion

It was concluded that the pedicled tensor fascia lata flap is a versatile, reliable, easy, and less time consuming procedure for the coverage of defects around trochanter, groin, lower anterior abdomen, perineum and ischial region.
